# An Anatomical Act for Georgia

**Published:** 1886-11

**Authors:** 


### AN ANATOMICAL ACT FOR GEORGIA.

We present in this number of THE JOURNAL an interesting letter from our Philadelphia correspondent touching this subject. The workings of the "Anatomical Act of Pennsylvania," as given by him, demonstrate clearly what we claimed in a previous number, that grave-robbing is not only prevented by such a law, but rendered impossible.

There seems to be little doubt that the next Legislature will enact such a law for our State.

The Atlanta *Evening Journal*, the only paper in the State that opposed the bill before the last Legislature, says: "The subject needs the prompt attention of our next Legislature, and legislation on this line should be enacted and enforced."

The *Christian Index*, one of the most influential religious papers in Georgia, says: "There should be a law giving to medical colleges all dead bodies *not claimed;* the colleges should be required to embalm them, and make no use of them for say three months, and be under obligations to give them up if claimed within that time, and be under further obligations to give the remains decent burial after dissection. Ample material could be provided for medical colleges in this way and nobody would be hurt by it. In the absence of such law the whole community is exposed to outrage. Yet the Legislature steadily refuses to give the people the protection for their dead which they have a right to demand. It is to be hoped that our next Legislature will yield to the dictates of reason, common sense and common humanity."

*The Wesleyan Christian Advocate*, an influential church paper, says : "The State of Georgia has chartered several medical colleges, and is supposed to favor the study of medicine and surgery, but the Legislature has never made any proper provisions for subjects for the dissecting rooms. Grave-robbing has therefore become a very extensive industry in this good old commonwealth. It is a sickening business and one that keeps the friends of the dead in constant and painful fear that the remains of their departed loved ones will be ruthlessly taken up, dumped into a cart and delivered during the hours of the night to the agent of some medical college. This question should be made one of urgency at the approaching session of the Georgia Legislature.

